# Tissue-resident memory-like ILCs: innate counterparts of T_RM_ cells

**DOI:** 10.1007/s13238-019-0647-7

**Published:** 2019-07-08

**Authors:** Xianwei Wang, Zhigang Tian, Hui Peng

**Affiliations:** 1 The First Affiliated Hospital of USTC, Division of Life Sciences and Medicine, University of Science and Technology of China, Hefei, 230001 China; 2 Division of Molecular Medicine, Hefei National Laboratory for Physical Sciences at Microscale, The CAS Key Laboratory of Innate Immunity and Chronic Disease, School of Life Sciences, University of Science and Technology of China, Hefei, 230027 China; 3 Institue of Immunology, University of Science and Technology of China, Hefei, 230027 China

**Keywords:** tissue-residency, innate lymphoid cells, immunological memory, T_RM_ cells

## Abstract

Innate lymphoid cells (ILCs) are defined as lymphocytes that lack RAG recombinase and do not express diverse antigen receptors; however, recent studies have revealed the adaptive features of ILCs. Mouse cytomegalovirus (MCMV)- and cytokine-induced memory natural killer (NK) cells circulate in the blood and are referred to as conventional memory NK cells. In contrast, virus- and hapten-induced memory NK cells, hapten-induced memory ILC1s, and cytokine-induced memory-like ILC2s exhibit long-term residency in the liver or lung, and are referred to as tissue-resident memory ILCs. Considering their similar migration patterns and memory potential, tissue-resident memory ILCs could be regarded as innate counterparts of resident memory T (T_RM_) cells. Both tissue-resident memory ILCs and T_RM_ cells share common characteristics in terms of dynamics, phenotype, and molecular regulation. The emergence of ILC memory expands the basic biology of ILCs and prompts us to re-examine their functions in disease progression. This review discusses the evidence supporting tissue-resident memory NK cells and other memory ILC subsets, compares them with T_RM_ cells, and highlights key unsolved questions in this emerging field.

## INTRODUCTION

Innate lymphoid cells (ILCs), including natural killer (NK) cells, ILC1s, ILC2s, ILC3s, and lymphoid tissue-inducer (LTi) cells, are a group of innate lymphocytes that confer early defense against pathogenic infections and tumor development (Cella et al., 2008; Cupedo et al., 2008; Sanos et al., 2008; Satoh-Takayama et al., 2008; Moro et al., 2009; Neill et al., 2010; Fuchs et al., 2013; Klose et al., 2014; Vivier et al., 2018). However, recent studies have uncovered the adaptive features of NK cells and other ILCs (O’Leary et al., 2006; Cooper et al., 2009; Sun et al., 2009; Peng et al., 2013a; Martinez-Gonzalez et al., 2016; Wang et al., 2018, 2019). Mouse cytomegalovirus (MCMV) infection induces the generation of memory Ly49H^+^ NK cells (Sun et al., 2009), and cytokine stimulation with interleukin (IL)-12/15/18 mediates memory-like NK cell formation (Cooper et al., 2009). MCMV- and cytokine-induced memory NK cells circulate in the blood and populate throughout the body. Herein, we refer to these cells as conventional memory NK cells (Table 1). Besides this type of memory NK cells, recently defined memory ILCs show long-term residency in peripheral tissue, but not in the circulation, and they are referred to as tissue-resident memory ILCs (Table 1). For example, hapten-induced memory NK cells and ILC1s are liver-resident (Paust et al., 2010; Peng et al., 2013a; Li et al., 2017; Wang et al., 2018), and IL-33-responsive ILC2s can acquire memory potential and persist in the lung (Martinez-Gonzalez et al., 2016). These new findings prompt us to re-examine the biology of ILCs and their roles in disease progression.

**Table 1:** Characteristics of tissue-resident memory ILCs and conventional memory NK cells

	Conventional memory NK		Tissue-resident memory ILCs		
	Ly49H^+^cNK	cNK	trNK	ILC1	ILC2
Tissue-residency	-	-	liver	LNs, liver	LNs, Lung
Antigen specify	MCMV-m157	IL-12/15/18 (non-specific)	Haptens, Influenza, VSV, HIV	Haptens	IL-33 (non-specific)
Recall responses	IFN-γ ↑ Cytotoxicity ↑ Expansion ↑	IFN-γ ↑	IFN-γ ↑ Cytotoxicity ↑	Inflammation ↑	IL-5 ↑ IL-13 ↑
Longevity	+	+	+	+	+

MCMV, mouse cytomegalovirus; cNK, conventional NK cells; trNK, tissue-resident NK cells; LNs, lymph nodes; VSV, vesicular stomatitis virus; HIV, human immunodeficiency virus

The system of memory T cells, including effector memory T (T_EM_) cells, central memory T (T_CM_) cells, and resident memory T (T_RM_) cells, is well established (Mueller et al., 2013; Schenkel and Masopust, 2014; Mueller and Mackay, 2015). The T_EM_ cells are abundant in non-lymphoid tissues; T_CM_ cells predominantly exist in secondary lymphoid organs, whereas T_RM_ cells are non-recirculating populations that persist in the infectious sites, such as the skin (Jiang et al., 2012; Mackay et al., 2013), intestine (Sheridan et al., 2014; Zundler et al., 2019), lung (Teijaro et al., 2011; Laidlaw et al., 2014), liver (Fernandez-Ruiz et al., 2016; Mackay et al., 2016), and brain (Wakim et al., 2010; Smolders et al., 2018). MCMV-induced conventional memory Ly49H^+^ NK cells have similarities with CD8^+^ T_EM_ cells in terms of migration patterns, dynamics, and molecular and epigenetic regulation (O’Sullivan et al., 2015; Lau et al., 2018; Rapp et al., 2018; Wu and Wang, 2018). In contrast, other memory ILCs share many common features with T_RM_ cells, particularly in their tissue-residency and regulation mechanisms. Thus, tissue-resident memory ILCs could be regarded as innate counterparts of T_RM_ cells. As conventional memory NK cells have been well summarized (O’Sullivan et al., 2015; Cerwenka and Lanier, 2016; Pahl et al., 2018; Rapp et al., 2018), in this review, we focus on discussing the evidence of tissue-resident memory NK cells and other ILC subsets, comparing memory ILCs and T_RM_ cells in detail, and updating future challenges in the field.

## TISSUE-RESIDENT MEMORY NK CELLS

**Figure 1:**
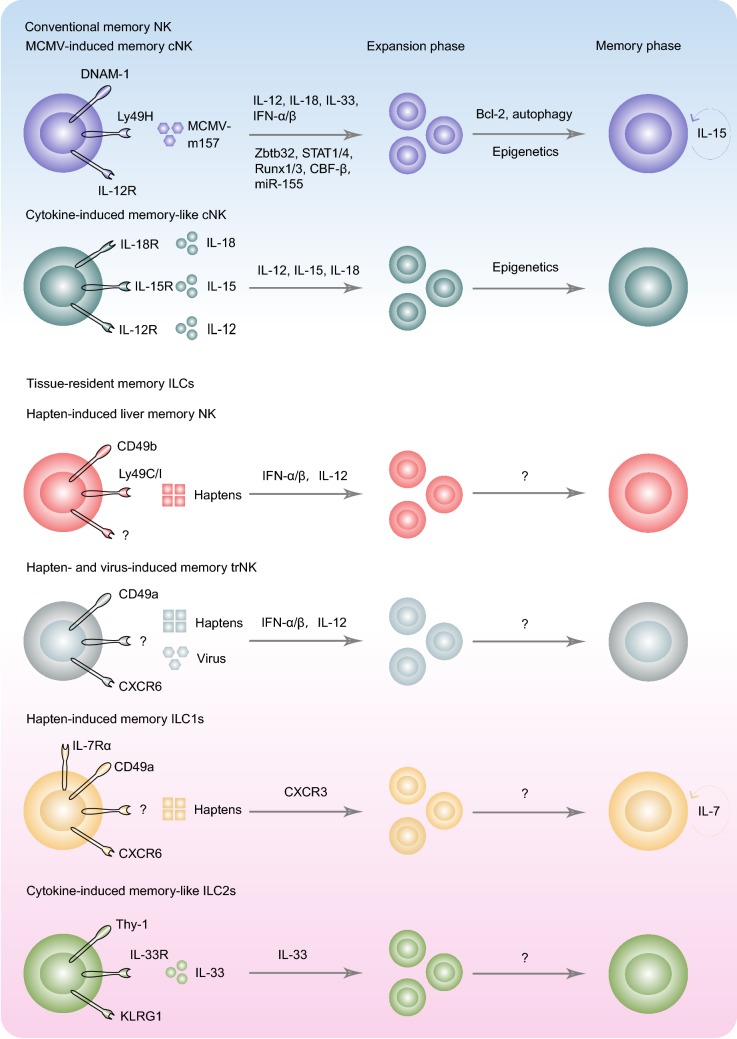
**Memory formation and long-term maintenance of tissue-resident memory ILCs and conventional memory NK cells**. Memory ILCs comprise MCMV- or IL-12/15/18-induced circulating populations, and hapten-, virus-, or IL-33-induced tissue-resident subsets. Antigen-specific cNK cells respond to MCMV via three signals, including recognition receptor Ly49H, co-stimulatory molecule DNAM-1, and pro-inflammatory cytokines. Effector Ly49H^+^ NK cells go through the expansion phase under the control of transcription factors, such as Zbtb32, STAT1/4, Runx1/3, and CBF-β. A small fraction of effector Ly49H^+^ NK cells can survive against apoptosis to generate a memory pool. Memory Ly49H^+^ NK cells circulate in the blood and populate throughout the body. Cytokine-induced non-specific memory cNK cells also show similar migration patterns. Apart from these circulating memory NK cells, hapten-induced memory NK cells and ILC1s exhibit long-term residency in the liver. CD49b^+^ cNK cells may recognize haptens or hapten-peptide complexes dependently on the Ly49C/I receptors. Inflammatory cytokines IFN-α/β and IL-12 drive the memory formation of hapten-specific liver NK cells. Of note, key factors contributing to the retention of memory CD49b^+^ cNK cells have not been determined. CD49a^+^CXCR6^+^ NK cells can mediate recall responses to different viruses and haptens; however, the associated recognition receptors remain largely unknown. Both CD49b^+^Ly49C/I^+^ and CD49a^+^CXCR6^+^ NK cells prefer to reside in the liver after memory formation. Haptens also induce memory IL-7Rα^+^ ILC1 generation in draining LNs. The LN-derived memory ILC1s selectively maintain their longevity in the liver via CXCR6 and IL-7. In addition, IL-33-experienced lung ILC2s exhibit higher responsiveness to IL-33 and IL-25, confirming their memory-like features

Owing to a lack of RAG recombinase, NK cells cannot generate diverse antigen recognition receptors, and are classified as innate lymphocytes. NK cells produce cytotoxic granules and interferon (IFN)-γ, conferring early defense against viral infections and tumor development. However, in the last decade, adaptive features of NK cells have been observed by several independent laboratories in different experimental systems, including MCMV m157 protein-, cytokine- and hapten-induced NK cell recall responses (O’Leary et al., 2006; Cooper et al., 2009; Sun et al., 2009). These outstanding findings advance the concept of NK cell memory. MCMV and cytokines induce conventional memory NK cells, whereas hapten-induced memory NK cells show long-term tissue residency in the liver (O’Leary et al., 2006; Paust et al., 2010). O’Leary and colleagues found that hapten-sensitized liver NK cells, but not splenic NK cells, can transfer memory to naïve mice in the contact hypersensitivity (CHS) model, revealing the “liver-restricted” properties of hapten-induced memory NK cells (O’Leary et al., 2006). Liver memory NK cells have a high level of CXCR6 expression, which is critical for their long-term homeostasis (Paust et al., 2010) (Fig. 1). Only liver CXCR6^+^ NK cells can mediate intense skin inflammation, suggesting that NK cells with memory potential are concentrated in the CXCR6^+^ populations (Paust et al., 2010). We have found that liver NK cells are heterogeneous populations, consisting of the tissue-resident CD49a^+^ subset and conventional CD49b^+^ populations (Peng et al., 2013a; Peng and Sun, 2017). The former accounts for approximately 50% of total liver NK cells and do not participate in the circulation; whereas the latter circulate in the blood (Peng et al., 2013a). Liver-resident NK cells develop dependently on the transcription factors T-bet and Hobit (Sojka et al., 2014; Mackay et al., 2016), in contrast to Eomes-dependent conventional NK (cNK) cells (Sojka et al., 2014); thus, they represent a distinct lineage from bone marrow-derived cNK cells. More interestingly, CD49a^+^ liver-resident NK cells highly express CXCR6, whereas CD49b^+^ cNK cells lack the expression of CXCR6 (Peng et al., 2013a; Wang et al., 2018). After skin sensitization with the hapten fluorescein isothiocyanate (FITC), a few FITC-positive cells can be found in the liver (Peng et al., 2013a). Although recognition receptors have not been determined, liver-resident CD49a^+^CXCR6^+^ NK cells may be sensitized in an antigen-specific manner. Adoptive transfer of hapten-sensitized CD49a^+^ liver-resident NK cells induces allergic skin inflammation, revealing the memory potential of liver-resident CD49a^+^CXCR6^+^ NK cells (Peng et al., 2013a) (Fig. 1). The unique microenvironment in the liver modulates tissue-resident memory NK cells, among which the ligands for CXCR6 and aromatic hydrocarbon receptor (AhR) signals are important for their memory potential and homeostasis (Paust et al., 2010; Zhang et al., 2016). CXCR6- or AhR-deficient liver-resident NK cells fail to recall specific haptens (Paust et al., 2010; Zhang et al., 2016). Moreover, AhR^−/−^ mice have decreased CD49a^+^ NK cell numbers, suggesting that AhR supports their normal development (Zhang et al., 2016). Of note, the skin contains a considerable number of CD49a^+^ tissue-resident NK cells (Sojka et al., 2014); whether these cells respond to haptens locally has not been determined.

Liver-resident memory NK cells can also recall viral antigens (Fig. 1). Paust and colleagues reported that liver CXCR6^+^ NK cells protect mice, which have been immunized with the influenza virus, vesicular stomatitis virus, or human immunodeficiency virus type 1, from second lethal challenges, revealing that liver-resident NK cells acquire memory potential against specific viruses (Paust et al., 2010). Consistent with these findings, another study has shown that adoptive transfer of influenza virus-vaccinated liver CD49a^+^ NK cells prolongs the survival of mice infected with a lethal dose of the influenza virus (Li et al., 2017). Although NKp46 on NK cells recognize hemagglutinin (HA) of the influenza virus (Arnon et al., 2004), HA is not required for influenza virus-driven memory NK cell generation (Paust et al., 2010). How liver CD49a^+^CXCR6^+^ NK cells discriminate different haptens and viruses remains a mystery. Another unsolved question is how viral infection in the lung induces the formation of antigen-specific memory cells in the liver. In addition, liver-resident NK cells can confer earlier protection against MCMV infection than conventional NK cells (Weizman et al., 2017). Liver-resident NK cells lack Ly49H expression, which recognizes MCMV m157 antigen; thus, these populations cannot respond directly to MCMV. Dendritic cell (DC)-derived IL-12 is required for liver-resident NK cell activation in the settings of MCMV infection (Weizman et al., 2017). Considering that cytokine IL-12/15/18 stimulation generates murine memory-like NK cells (Cooper et al., 2009), liver-resident NK cells may acquire non-specific cytokine-induced memory in MCMV infection. Despite these mysteries, these findings provide data for new strategies of vaccine development based on tissue-resident memory NK cells.

Following the discovery of murine liver-resident CD49a^+^ NK cells, several studies have confirmed the presence of liver-resident NK cells in humans. However, precise phenotypic and transcriptional features of human liver-resident NK cells are still confusing. Both the CD49a^+^Eomes^−^ subset and CD49a^−^CD49e^−^CXCR6^+^Eomes^hi^ subset have been identified as human liver-resident NK cells, which are absent in the peripheral blood; the former accounts for only a small fraction of total liver NK cells (0%–12%), whereas the latter accounts for nearly 50% (Marquardt et al., 2015; Cuff et al., 2016; Hudspeth et al., 2016; Stegmann et al., 2016; Aw Yeang et al., 2017) . In addition, cytokines including IL-2, IL-12, IL-15, and IL-18, can induce the conversion of peripheral CD49a^−^ NK cells into CD49a^+^ resident-like populations (Hydes et al., 2017). Moreover, the frequency of CD27^−^CD11b^−^ NK cells increases up to 40% in the liver of hepatocellular carcinoma (HCC) patients (Zhang et al., 2017). As mouse CD49a^+^ NK cells also substantially exhibit CD27^−^CD11b^−^ phenotype (Tang et al., 2016), HCC-induced CD27^−^CD11b^−^ NK cells may represent a resident population. Further studies should confirm the expression of CD49a and CXCR6 on human CD27^−^CD11b^−^ NK cells. Interestingly, a clinical study of liver transplants showed that donor Eomes^hi^ NK cells resided in recipient livers for up to 13 years, revealing the long-term renewal capability of Eomes^hi^ liver-resident NK cells (Cuff et al., 2016). Another study showed that CD49a^+^ liver-resident NK subsets can survive for 3 weeks *in vitro* (Marquardt et al., 2015). Thus, both human CD49a^+^ and CD49a^−^ liver-resident NK cells are long-lived populations, demonstrating their memory-like features. Of note, human CD49a^+^ liver-resident NK cells express NKG2C (Marquardt et al., 2015), which forms complexes with CD94 to recognize human cytomegalovirus (HCMV) UL40 peptides and drives the memory formation of NK cells (Hammer et al., 2018). In addition, cytokines, including IL-12, -15, and -18, have been reported to mediate memory generation of human NK cells (Cooper et al., 2009; Romee et al., 2012). It will be of great interest to explore whether HCMV and cytokines can induce memory responses of human tissue-resident NK cells in the liver.

Although CD49b^+^ cNK cells are considered as circulating lymphocytes, liver CD49b^+^ cNK cells have been reported to confer hapten-induced “liver-restricted” memory responses (van den Boorn et al., 2016; Wight et al., 2018) (Fig. 1). Pro-hapten monobenzone-induced CHS responses are primarily driven by memory CD49b^+^ cNK cells (van den Boorn et al., 2016). Monobenzone sensitization induces the activation of NLRP3 inflammasome in macrophages (van den Boorn et al., 2016). Then macrophage-derived IL-18 activates hapten-specific cNK cells, promoting their memory formation (van den Boorn et al., 2016). Details of the mechanisms involved in this process need further investigation. As pro-hapten monobenzone is metabolized in melanocytes to generate haptens, monobenzone-induced memory CD49b^+^ cNK cells display specific cytotoxicity against melanocytes, thereby mediating allergic skin inflammation (van den Boorn et al., 2016). In addition, monobenzone-induced memory CD49b^+^ cNK cells can effectively control B16 tumor development, revealing the clinical value of memory NK cells in tumor immunotherapies (van den Boorn et al., 2016). Like memory NK cells induced by other haptens, monobenzone-induced memory CD49b^+^ cNK cells are liver-resident populations, as evidenced by findings that monobenzone-sensitized liver CD49b^+^ cNK cells, but not their splenic counterparts, can confer CHS responses (van den Boorn et al., 2016). Previous studies have shown that NK cells with hapten-specific memory potential are concentrated in Thy-1^+^ or Thy-1^+^Ly49C/I^+^ fractions (O’Leary et al., 2006; Gillard et al., 2011; Majewska-Szczepanik et al., 2013). Interestingly, a recent study provided insights into Ly49C/I-dependent antigen recognition mechanisms of memory NK cells (Cooper, 2018; Wight et al., 2018) (Fig. 1). Wight and colleagues found that hapten-induced CHS responses were impaired in the *Rag1*^−/−^*Ly49*^KD^ mice, which exhibited reduced Ly49 receptor expression (Wight et al., 2018). Knock-in of Ly49I can rescue NK cell-mediated memory responses (Wight et al., 2018). Moreover, similar to haptens, Ly49C/I-sensitive peptides also induce NK cell-mediated CHS responses (Wight et al., 2018). This study raises the possibility that haptens may form complexes with Ly49C/I-sensitive peptides to generate complete antigens, which can be recognized by the Ly49C/I receptors on NK cells. In addition, molecules associated with homing and activation, such as CXCR6, CD62L, CD18, and NKG2D, play a role in liver NK cell-mediated memory responses (O’Leary et al., 2006). Among these surface molecules, Ly49C/I and CD62L are expressed on CD49b^+^ cNK cells at much higher levels than on liver-resident NK cells at steady state (Peng et al., 2013b; Wang et al., 2018); whereas CXCR6 shows an opposite expression pattern (Wang et al., 2018); Thy-1, CD18, and NKG2D are widely expressed on both subsets (Wang et al., 2018). As CD49b^+^ cNK cells lack CXCR6 and CD49a, the specific factors responsible for the tissue-residency of memory CD49b^+^ cNK cells in the liver remain unclear. Collectively, two parallel resident memory systems of liver-resident CD49a^+^CXCR6^+^ NK cells and conventional CD49b^+^Ly49C/I^+^ NK cells may exist in the liver after hapten sensitization.

Currently, the presence of CD49a^+^ tissue-resident NK cells has been confirmed in multiple organs, such as the skin, uterus, and salivary glands (Sojka et al., 2014; Cortez et al., 2016). Immune cells contribute to the homeostasis of adult uterus, among which tissue-resident NK cells comprise the dominant populations (Fu et al., 2017; Andreotti et al., 2018; Filipovic et al., 2018; Sojka et al., 2018). In mice, more than 60% of the uterine NK subsets express CD49a; in humans, uterine NK cells mostly exhibit CD56^brihgt^CD49a^+^ phenotype (Fu et al., 2017). More recently, human uterine-resident CD56^bright^NKG2C^hi^ NK cells have been shown to appear in multigravid women, but not in primigravid women; thus, this population is referred to as pregnancy trained decidual NK (PTdNK) cells (Gamliel et al., 2018). Human PTdNK cells secrete IFN-γ and VEGFα; the latter supports vascularization and promotes fetal development (Gamliel et al., 2018). Repeated pregnancies make the *IFNG* and *VEGFA* locus more accessible, resulting in increased production of IFN-γ and VEGFα in subsequent pregnancies, a process similar to memory-like recall responses (Gamliel et al., 2018). As lacking certain antigens, tissue-resident memory-like PTdNK cells should be classified as non-specific memory-like NK cells. Moreover, IL-15 and activating receptor HLA-G are considered as triggers that generate memory-like potential of PTdNK cells (Gamliel et al., 2018). Human conventional memory NK cells, induced by HCMV and cytokines, have been well documented (Lopez-Verges et al., 2011; Romee et al., 2012), and share common features and unique characteristics with uterine-resident memory-like NK cells. Conventional memory NK cells circulate throughout the body, whereas uterine memory-like NK cells exhibit features of long-term tissue-residency. Phenotypically, HCMV UL40 peptide-induced conventional memory NK cells are characterized by the CD56^dim^NKG2C^hi^CD57^+^ phenotype (Lopez-Verges et al., 2011; Hammer et al., 2018); cytokine-induced memory-like NK cells are characterized by the CD56^bright^NKG2A^+^CD69^+^ or CD56^dim^NKG2A^+^CD69^+^ phenotype (Romee et al., 2012); and uterine-resident memory-like PTdNK cells show the CD56^bright^NKG2C^high^ phenotype (Gamliel et al., 2018). It should be noted that naïve uterine-resident NK cells lack NKG2C expression; thus, NKG2C may be a reliable marker to distinguish uterine memory-like NK cells from naïve cells. In addition, HCMV infection induces generation of memory NK cells in an antigen-specific manner; whereas both cytokine-induced and pregnancy-trained memory-like NK cells are non-specific.

## TISSUE-RESIDENT MEMORY ILC1S

All helper ILCs, including ILC1s, ILC2s, and ILC3s, are tissue-resident innate populations (Gasteiger et al., 2015). ILC1s are defined as T-bet dependent IFN-γ/TNF-producing subsets, which lack cytotoxic abilities and confer earlier host defense than cNK cells (Artis and Spits, 2015; Eberl et al., 2015; Weizman et al., 2017; Vivier et al., 2018). However, the boundary between ILC1s and tissue-resident NK cells is still blurred. In addition to tissue-resident markers, IL-7Rα might be an important marker for helper ILC1s (Diefenbach et al., 2014; Klose et al., 2014). Our recent study has uncovered the adaptive features of IL-7Rα^+^ ILC1s in the CHS model (Fig. 1). Hapten sensitization initiates the recruitment of IL-7Rα^+^ ILC1s into skin-draining lymph nodes (LNs) in a CXCR3-dependent fashion (Wang et al., 2018). LN ILC1s exhibit the IL-7Rα^+^Thy-1^+^CD62L^+^CD18^+^NKG2D^+^CD49a^+/−^CXCR6^+/−^ Ly49C/I^−^ phenotype (Wang et al., 2018). IL-7Rα^+^ ILC1s are primed within 48 h post sensitization and acquire memory potential against haptens at 72 h in draining LNs (Wang et al., 2018). As IL-7Rα^+^ ILC1s lack Ly49C/I, the manner in which ILC1s recognize different haptens remains unclear. Memory ILC1s exit draining LNs via sphingosine-1-phosphate receptor-1 (S1PR1) and selectively reside in the liver via CXCR6-CXCL16 interaction (Wang et al., 2018). Moreover, IL-7 has been shown essential for memory ILC1 longevity. Liver sinusoidal endothelial cells (LSECs) and hepatocytes may be sources of IL-7 (Wittig et al., 2010; Liang et al., 2012). As IL-7Rα deficiency does not affect expression of the anti-apoptosis protein BCL2 in ILC1s (Robinette et al., 2017), IL-7 may support long-lived IL-7Rα^+^ ILC1s in BCL-2-independent mechanisms. Besides BCL-2, IL-7Rα signaling also induces intrinsic fatty acid oxidation (FAO) to support the long-term survival of T_RM_ cells (Cui et al., 2015). As recent studies have revealed a critical role of metabolism in conventional and adaptive NK cell function and survival (Cichocki et al., 2018; Cong et al., 2018; O’Brien and Finlay, 2019), whether the IL-7-FAO axis contributes to memory ILC1 longevity needs further research. Overall, the liver provides suitable niches, with an enrichment of CXCL16 and IL-7, for the long-term residency of memory ILC1s.

Allergic contact dermatitis (ACD) is a common occupational skin disease caused by allergens, such as nickel and house dust mite allergens, which accounts for 20% of the entire work-related health burden (Kaplan et al., 2012). T cells are traditionally defined as key players in ACD progression. Interestingly, human CD56^bright^CD16^−^CD62L^−^ NK cells have been found to accumulate in the skin lesions of ACD patients (Carbone et al., 2010). These infiltrated NK cells produce high levels of IFN-γ and TNF, exhibiting ILC1 characteristics. Although the expression of CD49a and CXCR6 has not been investigated, increased CD56^bright^ NK cells highly express CCR5 (Carbone et al., 2010), a chemokine receptor essential for the tissue-residency of human liver NK cells (Hudspeth et al., 2016), suggesting that allergen reactive CD56^bright^ NK subsets may be skin-resident populations. Moreover, skin CD56^bright^ NK cells from ACD patients are CXCR3 positive (Carbone et al., 2010), which is critical for the memory generation of liver-resident IL-7Rα^+^ ILC1s (Wang et al., 2018), implying that human skin-resident NK cells or ILC1s may be involved in allergen-induced memory responses. However, human skin NK cells from ACD patients have no recall responses to the nickel allergen (Carbone et al., 2010). Recent studies have raised the possibility that Ly49C/I may interact with sensitive peptide-hapten complexes (Wight et al., 2018); thus, human NK cells may recognize nickel-self protein complexes, but not nickel ions. Collectively, some studies have implied that human tissue-resident NK cells or ILC1s may also have adaptive features. Humanized mouse models might be useful tools to confirm this hypothesis.

## TISSUE-RESIDENT MEMORY-LIKE ILC2S

ILC2s are traditionally classified as GATA3- and RORα-dependent, IL-4-, IL-5, and IL-13-producing innate tissue-resident lymphocytes, which are key players in the early stage of fungal infection and allergen-induced type II responses (Moro et al., 2009; Neill et al., 2010; Artis and Spits, 2015; Eberl et al., 2015; Vivier et al., 2018). Recently, Martinez-Gonzalez and colleagues revealed the immunological memory of lung ILC2s (Martinez-Gonzalez et al., 2016; Martinez-Gonzalez et al., 2017) (Fig. 1). In the papain- or IL-33-induced allergic inflammation model, lung-resident ILC2s progressively go through the expansion, contraction, and stable memory phases (Martinez-Gonzalez et al., 2016). Lung-resident ILC2s receive initial activating signals via IL-33R, proliferate locally and produce amounts of IL-5 and IL-13 (Martinez-Gonzalez et al., 2016). Naïve ILC2s turn into effector state and attain their peak at day 6; the number of these effector ILC2s then decline and a small population survives to generate the memory pool (Martinez-Gonzalez et al., 2016). Re-stimulation of memory-like ILC2s by IL-33 leads to higher levels of IL-5 and IL-13 production, revealing the recall capacity of lung-resident ILC2s (Martinez-Gonzalez et al., 2016). Moreover, memory-like ILC2s show similar gene signatures with memory CD8^+^ T cells. Of note, naïve ILC2s express low levels of IL-25R, whereas IL-33-primed ILC2s exhibit elevated IL-25R expression (Martinez-Gonzalez et al., 2016). Secondary challenge with various unrelated antigens also induce vigorous type II responses of memory-like ILC2s (Martinez-Gonzalez et al., 2016). In addition, IL-33-induced IL-5^+^IL-13^+^ ILC2s exhibit long-term residency in mediastinal lymph nodes (mLNs) (Martinez-Gonzalez et al., 2016), which implies that mLN-resident ILC2s may also have adaptive features. The discovery of tissue-resident memory-like ILC2s sheds new light on the roles of innate immune cells in chronic allergic inflammation.

## COMPARISON OF TISSUE-RESIDENT MEMORY ILCS and T_RM_ CELLS

In addition to T_EM_ and T_CM_ cells, T_RM_ cells have emerged as the third type of memory T cells (Mueller et al., 2013; Schenkel and Masopust, 2014; Mueller and Mackay, 2015). T_RM_ cells are generated at infectious sites and persist locally even after pathogen clearance. MCMV- and cytokine-induced conventional memory NK cells exhibit similar migration patterns with T_EM_ cells, which populate throughout the body; whereas recently defined tissue-resident memory ILCs mirror the generation and maintenance of T_RM_ cells. Virus- and hapten-induced memory CXCR6^+^CD49a^+^ NK cells and IL-7Rα^+^ ILC1s selectively reside in the liver (Paust et al., 2010; Peng et al., 2013a; Wang et al., 2018). IL-33-induced memory-like ILC2s show long-term residency in the lung (Martinez-Gonzalez et al., 2016). In this section, we discuss unique properties and common features of tissue-resident memory ILCs and T_RM_ cells.

### Antigen specificity

Memory T cells recall specific antigens depending on a diverse T-cell receptor (TCR) repertoire, whereas NK cells and other ILCs only express germline-encoded recognition receptors. cNK cells that remember specific antigens are well characterized in the CMV infection model, including murine Ly49H recognition of MCMV m157 protein (Brown et al., 2001; Arase et al., 2002) and human NKG2C recognition of HCMV UL-40 peptides (Hammer et al., 2018) (Fig. 1). Although liver-resident NK cells can specifically respond to viruses and haptens, the manner in which these cells distinguish different antigens remains largely unknown. A recent study revealed the critical role of Ly49C/I in hapten recognition of liver CD49b^+^ NK cells (Fig. 1). Interactions between Ly49C/I and sensitive peptides possibly occur in response to hapten sensitization (Wight et al., 2018); however, CXCR6^+^CD49a^+^ NK cell subsets lack Ly49C/I expression (Wang et al., 2018). Although NKp46 on NK cells can recognize the HA antigen of the influenza virus (Arnon et al., 2004), Paust and colleagues demonstrated that NKp46 did not participate in influenza infection-induced memory responses (Paust et al., 2010). In addition, ILCs can acquire memory-like potential in the absence of certain antigens, for example, memory-like ILC2s can recall the cytokine IL-33 (Martinez-Gonzalez et al., 2016). Cytokine-induced memory-like cells were firstly reported by Yokoyama laboratory (Cooper et al., 2009) and are defined as non-specific memory. Whether cytokine receptors on ILC1s and ILC3s can mediate similar recall responses has not been determined. Since the activating and inhibitory receptors required for ILCs to recognize specific and non-specific antigens are limited, ILCs are apparently unable to remember diverse pathogenic infections in nature.

### Phenotype

Expression of the lectin CD69 and the integrin CD103 is defined as the most common markers of T_RM_ cells (Mueller et al., 2013; Schenkel and Masopust, 2014; Mueller and Mackay, 2015). Interactions between CD69 and S1PR1 results in S1PR1 internalization and degradation; thus, CD69 functions as a suppressor of S1PR1 (Shiow et al., 2006; Mackay et al., 2015a). Reduced expression of S1PR1 limits the egress of lymphocytes into the circulation. Furthermore, CD103 can bind to E-cadherin on epithelial cells, and thereby lead to the retention of lymphocytes in local tissue (Cepek et al., 1994). In cases of CD103 deficiency, the numbers of T_RM_ cells are decreased in many organs, such as the skin (Mackay et al., 2013) and small intestine (Sheridan et al., 2014). Notably, CD69 and CD103 co-expression cannot mark all types of T_RM_ cells. For example, *Yersinia pseudotuberculosis* infection induces the generation of CD103^−^ T_RM_ cells (Bergsbaken and Bevan, 2015). Regarding ILCs, CD69 is expressed on tissue-resident NK cells and intestine ILC1s (Tang et al., 2016), whereas CD103 is expressed at low or undetermined levels on ILC subsets (Vivier et al., 2018). The integrin CD49a, which binds to type IV collagen, is a classic marker of tissue-resident NK cells and intestine ILC1s (Peng et al., 2013a; Sojka et al., 2014; Tang et al., 2016). However, our unpublished data show that liver-resident NK cells exhibit normal frequencies and numbers in CD49a-deficient mice, suggesting that CD49a alone is not sufficient for NK cell retention in the liver. Interestingly, recent studies have revealed that CD49a expression can also mark T_RM_ cells in the skin and liver (Mackay et al., 2016; Cheuk et al., 2017). In addition, elevated IL-25R expression on allergen-experienced ILC2s indicates that IL-25R may be a reliable marker for memory-like ILC2s. Whether these memory-like ILC2s express CD69, CD103, or CD49a needs further research.

### Location and dynamics

Upon exposure to pathogens and non-infectious antigens, both tissue-resident ILCs and T cells undergo expansion, contraction, and stable memory phases. After hapten sensitization, IL-7Rα^+^ ILC1s are recruited to draining LNs in a CXCR3-dependent fashion at 24 h, and peak at 48 h. Effector ILC1s then exhibit a dramatic loss in numbers, independent of apoptosis, and eventually generate the memory cell pool at 72 h (Wang et al., 2018). Although liver CD49b^+^Ly49C/I^+^ NK cells also respond to specific haptens, the detailed dynamics have not been described. Upon encountering IL-33, lung-resident ILC2s have a 50-fold expansion at day 6, and differentiate into effector cells locally. These effector ILC2s then undergo contraction, and a small fraction of ILC2s can survive and last for at least 160 days (Martinez-Gonzalez et al., 2016). In humans, adaptive CD56^bright^NKG2C^+^ NK cells are rare in the uterus, while these subsets can be induced by HLA-G and IL-15 during pregnancy (Gamliel et al., 2018). Whether similar NK cell subsets exist in mice has not been explored; nevertheless, pregnancy also induces the expansion and contraction of uterine-resident NK cells in mice and humans (Fu et al., 2017; Filipovic et al., 2018; Sojka et al., 2018).

### Inflammatory factors drive tissue-resident memory cell formation

Before memory cell formation, three signals are required for T cell activation, including antigen-recognition TCR signals, co-stimulatory signals, and inflammatory cytokine signals. Although antigen-recognition receptors and co-stimulatory molecules on memory ILCs have not been well determined, pro-inflammatory cytokines, such as IFN-α, IFN-γ, IL-12, IL-25, and IL-33 have been shown to be essential during memory ILC formation (Majewska-Szczepanik et al., 2013; Martinez-Gonzalez et al., 2016) (Fig. 1). Majewska-Szczepanik and colleagues reported that hapten-sensitized liver mononuclear cells from IFN-α^−/−^, IFN-γ^−/−^, or IL-12^−/−^ donors fail to transfer memory responses to naïve hosts, suggesting that inflammatory signals drive liver-resident memory NK cell formation (Majewska-Szczepanik et al., 2013). Similarly, in *Yersinia* infection, intestinal monocyte/macrophage-derived IL-12 and IFN-β promote CD103^−^CD69^+^ T_RM_ cell differentiation via negative regulation of CD103 expression (Bergsbaken et al., 2017). Furthermore, IL-33 is a potent stimulator of ILC2 cell activation (Salimi et al., 2013; Weiskirchen and Tacke, 2017; Tan et al., 2018). Martinez-Gonzalez and colleagues revealed the critical role of IL-33 in memory-like ILC2 formation. IL-33-experienced lung ILC2s acquire recall capacity for IL-33 or unrelated-allergens (Martinez-Gonzalez et al., 2016). Interestingly, IL-33 also drives T_RM_ cell formation by mediating the downregulation of transcription factor krüppel-like factor 2 (KLF2) (Skon et al., 2013). As the target gene of KLF2, S1PR1 determines the fate of memory cell traffic (Skon et al., 2013). In addition, inflammatory chemokines are also involved in the generation of resident memory ILCs and T_RM_ cells. Tissue-resident NK cells and ILC1s highly express CXCR3. In CXCR3 deficient mice, IL-7Rα^+^ ILC1s fail to be recruited into LNs and lose their capacity to recall haptens (Wang et al., 2018). Similarly, CD103^−^ T_RM_ cells also exhibit high levels of CXCR3 expression. CXCR3-dependent localization in inflamed areas of infected tissue is critical for CD103^−^CD69^+^ T_RM_ cell differentiation (Bergsbaken and Bevan, 2015). Another chemokine receptor, CXCR6, has been defined as a key regulator of the homeostasis and tissue residency of memory NK cells and IL-7Rα^+^ ILC1s (Paust et al., 2010; Wang et al., 2018). Zaid and colleagues also found that CXCR6, but not CXCR3, was responsible for skin-resident CD103^+^CD69^+^ T_RM_ cell formation and retention (Zaid et al., 2017).

### IL-7 and IL-15 support the long-term maintenance of tissue-resident memory ILCs and T cells

Common γ (γC) chain cytokines, IL-7 and IL-15, are well established as homeostatic factors for the longevity of T_CM_ and T_EM_ cells (Mueller et al., 2013). Several studies have also confirmed the contribution of both cytokines to the long-term maintenance of T_RM_ cells. Hair follicle-derived IL-7 and IL-15 are necessary for skin-resident memory CD4^+^ and CD8^+^ T cell homeostasis, as evidenced by findings that hapten-sensitized T cells from IL-7- or IL-15-deficient mice mediate impaired CHS responses (Adachi et al., 2015). Another study reported that blockade of IL-15 and IL-15R results in decreased antigen-specific CD103^+^ and CD103^−^ T_RM_ cell numbers in multiple organs, revealing the critical role of IL-15 in T_RM_ cell longevity (Mackay et al., 2015b). Similarly, long-term blockade of IL-7Rα in Rag1^−/−^ mice can reduce skin inflammation in the hapten-induced CHS model, suggesting that IL-7 is required for the longevity of liver-resident memory IL-7Rα^+^ ILC1s (Fig. 1). However, IL-7Rα deficiency does not affect BCL-2 expression in ILCs (Robinette et al., 2017); thus, BCL-2-independent mechanisms may play a role in IL-7-mediated memory ILC1 longevity. Although suitable levels of IL-15 is reported necessary for the formation and maintenance of MCMV-induced conventional memory Ly49H^+^ NK cells (Firth et al., 2013; Kamimura and Lanier, 2015) (Fig. 1), whether tissue-resident memory NK cells and other ILCs need IL-15 remains unclear. Overall, tissue-resident memory ILCs and T cells share similar mechanisms to maintain their long-term survival.

## CONCLUSIONS AND FUTURE PERSPECTIVES

Over the past 10 years, many studies about ILCs have been focused on their innate functions in pathogenic infection, inflammation, and tumor surveillance. However, accumulating evidence has revealed the adaptive features of ILCs and supports the concept of ILC memory. Two types of memory ILCs, including circulating memory NK cells and tissue-resident memory ILCs, have been confirmed by independent laboratories using different experimental systems in both mice and humans. Apart from CMV- and cytokine-induced conventional memory NK cells, resident memory NK cells and other ILCs exhibit long-term residency in the liver, uterus, and lung. Liver-resident NK cells can confer memory responses to viruses and haptens. Human adaptive CD56^bright^NKG2C^+^ NK cells are trained in the uterus by repeated pregnancies. IL-7Rα^+^ ILC1s acquire memory potential against haptens and selectively reside in the liver for long-term survival. Lung-resident ILC2s show recall capacities to the cytokine IL-33. It is becoming increasingly clear that tissue-resident memory ILCs may be innate counterparts of T_RM_ cells. They share common features, for example, similar location and dynamics, expression of resident markers, inflammatory factors driving memory cell formation, and IL-7 and IL-15 supporting their longevity. Along with a deeper understanding of memory ILCs, more detailed comparisons should be summarized.

In the emerging field of tissue-resident memory ILCs, several open questions remain unsolved. For example, besides the uterus, do resident memory NK cells and other ILCs exist in other human tissues? Do murine and human ILC3s have antigen-specific or non-specific memory potential? Do tissue-resident memory ILCs and T cells co-operate in the same cases? How do NK cells and ILC1s recognize different viruses and haptens? Which molecules are responsible for the tissue-residency of memory ILCs? How can the memory features of ILCs be used as powerful tools in clinical therapies, such as vaccine development? Despite many unsolved puzzles, the emergence of tissue-resident memory ILCs prompts us to re-examine the functions of ILCs in disease progression. Such new knowledge will deepen our understanding about the biology of ILCs and contribute to novel therapies in human disease.
